# The Appointment of State Boards of Medical and Dental Examiners

**Published:** 1901-07

**Authors:** William Carr

**Affiliations:** New York City


### THE APPOINTMENT OF STATE BOARDS OF MEDICAL AND DENTAL EXAMINERS.[1]

BY DR. WILLIAM CARR, NEW YORK CITY.

NOT many years ago it seemed to the majority, as it now seems to some of those interested in the advancement of medical and dental education, that diplomas from chartered colleges or schools gave sufficient assurance that their possessors were qualified to practise in the general or special fields of medicine in which degrees had been conferred upon them, and that the diplomas granted served as an adequate standard for license. This view assumed that instruction in such schools was faithfully given, by competent teachers, during a period of sufficient length to qualify students for the commencement of their careers, and also assumed that diplomas were conferred only upon those who, at the completion of such a period of study, had demonstrated, upon examination, their qualifications. It also assumed that charters would not be recklessly granted to professional schools, and that such charters would be revoked upon proof of their abuse. Experience has demonstrated the fallaciousness of such assumptions. Legislatures have passed incorporating acts most improvidently. So-called colleges have been created at the solicitation of charlatans having no other purpose in view than the manufacture and sale of parchments conferring degrees in absentia, without any safeguard or State supervision. The Buchanan and Delavan diplomas are

---

[1] Abstract of paper read before the American Medical Association, Section on Stomatology, St. Paul, Minn., June 4, 5, 6, and 7, 1901.

fair types of the product of these factories, which became a stench in the nostrils of decent men.

An attempt was made to remedy this evil, so far as it affected medical license in the State of New York, by a provision requiring that diplomas granted outside the State should be endorsed by our own schools. But this plan proved of little avail. Not only did respectable colleges endorse fraudulent and valueless papers, but the diploma business was carried on by certain of our native corporations.

It therefore became necessary to do away altogether with the diploma standard, for the reason that it could not be higher than that of the poorest institution authorized to confer a degree.

The plan of creating State examination boards, with power to supervise licenses, next suggested itself. At the commencement of the experiment it seemed to those resorting to the plan that to empower these boards to list the "reputable" schools and to accord to their diplomas only the licensing power, would meet the needs of the situation. In many jurisdictions such listing is still the chief function of the State board, and diplomas of the colleges classified as reputable afford therein a license to practise. But this plan has not proved successful, and the refusal of State boards to list certain of the so-called colleges has given rise to no small amount of litigation, especially in the West, where the right of a State board to exclude graduates of schools refused listing by the board has been vigorously attacked in mandamus proceedings.

In twenty-three States, however, the diplomas of even the best schools no longer afford a license to those upon whom they are conferred, but only serve as one of the qualifications of candidates for examination before State boards. This separation of the licensing power from the diploma has proved beneficial to the really reputable schools, some of whom strenuously opposed it. When the diploma constituted a license, faculties seemed to have been swayed, insensibly, perhaps, by a feeling that the payment of fees on the part of a student, especially if he were somewhat diligent, established a contract right on his part to receive collegiate authority to practise upon the public, and, as a consequence, the standard of education was low, compared with the standard of to-day. With the abolition of this right to license, which was only an apparent, not a real privilege, the value of a diploma was presently perceived to be proportionate to the reputation of the institution conferring it, which, in turn, was incited by self-interest as well as love of scholarship to raise its standard and confer its degree only upon those earning it. The period of study has been extended, and if the medical and dental laws have accomplished no other result than the raising of college standards, they find in that alone their ample justification.

The examination of candidates for license being vested in a State board, it is of the first importance that such a body should be properly constituted. In the first place, it goes without saying that its members should not only be honest and competent, but independent of extraneous influences, and with eyes single to the performance of their sole duty,—namely, the admission to the profession of men competent to practise and the exclusion of incompetents from its ranks. In order to command respect and authority, it is equally clear that the board should be composed of men of standing in the community and in their profession. Its members should be abreast of the science of the day in its application to their calling, for the rule of liability in malpractice cases takes into consideration the advanced state of science. Without being identified with teaching bodies, these examiners should be conversant with the arts of instruction and examination, able to frame questions of a nature to elicit the candidate's knowledge, and ascertain his ability to apply it. Unless the minds of the examiners and of the examined meet, unless each understands the other's aim, examinations are fruitless, impediments rather than aids.

Again, the office of examiner should be, and is, when proper tests are regarded in filling it, a post of honor. For that very reason it should never be filled by one whose appointment is not made because of his competency to perform the duty with which he is to be charged, but for the sake of conferring honor upon the incumbent. It is but natural that the post should be sought for the honor it confers, and yet a conscientious man, apprehending and purposing to perform faithfully its duties, will be slow to seek it. The position of an examiner upon a negligent or incompetent board is the reverse of honorable, and no man of professional pride and standing would suffer himself to be appointed to such a body.

Unhappily, there have been instances of late—let us be thankful that they did not occur, and could not have occurred, under the system adopted in the State of New York—of the appointment for personal and political reasons of conspicuously unfit boards. These appointments have been made through the heads of political divisions of the country, and it is but fair to presume that the successful candidates sought the places rather through political influence than by demonstration of their own fitness.

In our State of New York such scandals are not possible. I do not say this to praise our own State board, or to claim for it especial excellence on account of the individuals who compose it. To do so would merely be an exhibition of bad taste, and I certainly have no desire to under-estimate the many excellent, able, and learned men who honor the examining boards of other States. But what I mean to say is, that those very men are wronged and handicapped when the appointing power, for political reasons, associates them with men of lesser qualifications, and this is what has happened elsewhere, but cannot happen in the State of New York, for these reasons.

The University of the State of New York is not a teaching body, nor yet a political body, but is created for purposes of examination and supervision. It has been assailed and criticised from time to time by politicians who would abolish it, and thereby make way for some substitute which would afford patronage through themselves. Fortunately such efforts have hitherto failed, and whatever other criticism may have been made upon the Board of Regents, we have yet to hear any one suggest that its actions have been animated by political or partisan motives.

I sincerely hope that the day is not far distant when each State will have a board of regents, possessing the powers of the regents of the University of the State of New York, or, as suggested by President Henry Wade Rogers, that "there should be established in each State a council of education, which should be invested with powers similar to those of the regents of the University of the State of New York, and it should be composed of the most eminent men of the State, without any reference to political considerations. No degree-conferring power or no degree-conferring institution should be incorporated without the approval of the Council of Education."

With the University lies the power of appointing our boards of medical and dental examiners from candidates nominated by the State societies. The dental board is divided into four classes, the term of one class expiring on the 31st day of July in each year. Prior to the second Tuesday in June of each year the State society is empowered to nominate from its membership twice as many candidates for filling vacancies to occur on the board as there shall be such vacancies, and from these nominees the regents appoint.

Thus it is sought not only to remove the constitution of our examining board from State politics, but to take it out of professional politics. The State society, the purpose of whose incorporation is to assemble together men to whom the interest of the profession, its advancement, and honor are dear, and membership in which is open to all who are of good standing in the profession and chosen from their districts as representative men, has power to say to the regents, "We submit to you the following names of men who, in the opinion of the dental profession throughout the State, are fit and worthy to represent it. But we cannot appoint, we cannot by intrigue constitute the board; that must be done by the regents, and with them rests the responsibility for ill-advised selection, limited only by our responsibility in submitting poor material for their choice." Thus there is a double responsibility created: we are responsible for our nominees, and the regents for their appointments; and thus it is that we have removed, so far as human ingenuity could do so, the constitution of our board from the hands of the politicians; and if I have spoken of our system of licensing dentists as good, I have still spoken less strongly than Dr. Allen, secretary of the advisory committee of the National Association of Dental Faculties, who, writing under date of November 15, 1899, for "A Comprehensive Report from the New York Examiners," was kind enough to say of the body to which he belonged, "The entire committee regard the New York dental law as the best in the country." (See Regents' Bulletin 9, February, 1900, Dentistry, page 751, note.)

Before the board thus constituted come the candidates for license, after satisfying the Board of Regents that they have been graduated from the professional schools after receiving the proper preliminary education. But the examiners do not know the candidates who appear before them, or the schools from which they come. To guard against any possible favoritism, candidates are examined by number, and it has even happened that men have appeared before the board who have slipped by the outposts, and have passed the professional examination without having satisfied the preliminary requirements, with the result that their success in passing the State board has yet failed to license them to practise within the State of New York, by reason of their failure to comply with the preliminary requirements.

The success of our system, therefore, depends upon the existence in the State of New York of our University, which, while controlling all educating bodies, does not itself possess a teaching faculty; thereby have we been able to keep our board out of politics and have been able to create a double responsibility for its appointments.

That we have excluded from the profession all incompetents would be claiming to be something more than human; what we can safely maintain, however, is that we have guarded the entrance to the profession not only with sentinels, but with watchmen over the sentinels.

